# Ultrasonographic measurements of the inferior vena cava diameter in newborns: is it a useful tool for choosing an umbilical venous catheter?

**DOI:** 10.3389/fped.2023.1268622

**Published:** 2023-11-17

**Authors:** Francesca Galdo, Antonella Trappan, Francesca Cossovel, Carmen Rodriguez-Perez, Luca Ronfani, Paolo Montaldo, Cristina Bibalo, Laura Travan, Francesco Maria Risso

**Affiliations:** ^1^ Neonatal Intensive Care Unit, Institute of Child and Maternal Health, IRCSS Burlo Garofolo, Trieste, Italy; ^2^ Department of Medicine, University of Trieste, Trieste, Italy; ^3^ Neonatology and Neonatal Intensive Care Unit, ASST Spedali Civili, Ospedale Dei Bambini, Brescia, Italy; ^4^ Clinical Epidemiology and Public Health Research Unit, Institute for Maternal and Child Health—IRCCS “Burlo Garofolo,” Trieste, Italy; ^5^ Department of Neonatal Intensive Care, Università degli Studi della Campania Luigi Vanvitelli, Naples, Italy; ^6^ Department of Brain Sciences, Centre for Perinatal Neuroscience, Imperial College, London, United Kingdom; ^7^ Azienda Sanitaria Universitaria Giuliano Isontino, Trieste, Italy

**Keywords:** umbilical catheters, measurements, diameter, vena cava, ultrasound, newborn

## Abstract

**Objectives:**

The primary outcomes of this study were to evaluate the diameters of the inferior vena cava (IVC) in a cohort of newborns and the correlation between newborn weight and IVC diameter. The secondary outcome was to evaluate the concordance between the measurements performed by the two investigators.

**Methods:**

Two blind examiners performed an ultrasonographic (US) evaluation of the IVC diameter in neonates with a weight ranging from 2 to 4 kg. The exclusion criteria included hemodynamic instability, known vascular malformations, and major congenital malformations.

**Results:**

A total of 143 neonates were enrolled between June 2019 and January 2021. All the US examinations were performed in the first 3 days of life. After dividing the patients into two groups according to their weight at the time of examination (2.0–2.99 kg and 3.0–4.0 kg), the median IVC diameters measured by examiner 1 were 3.1 mm (interquartile range 2.8–3.4) and 3.4 mm (interquartile range 2.9–3.8) (*p* = 0.003) for the two groups, respectively. The median IVC diameters measured by examiner 2 were 3.1 mm (interquartile range 2.6–3.3) and 3.3 mm (interquartile range 2.8–3.8) (*p* = 0.004) for the two groups, respectively. The intraclass correlation coefficient was 0.93 (95% CI: 0.90–0.95).

**Conclusion:**

The IVC diameter values varied widely from 1.2 to 5.2 mm in newborns weighing 2–4 kg, and a low correlation between newborn weight and IVC diameter was found, so measuring IVC diameter may be a recommended step prior to inserting a umbilical venous catheter (UVC). The concordance between operators was good. We contemplated that the IVC diameter could be a potentially useful tool to identify the most appropriate UVC, thus reducing the risk of catheter-related thrombosis.

## Introduction

Umbilical venous catheters (UVCs) are commonly used in NICUs to manage high-risk newborns in administering parenteral nutrition, hypertonic solutions, medications, and exchange transfusion and providing emergency intravenous access. Not only preterm babies but also sick term babies may require UVCs.

Although catheter insertion could be easily performed, a neonatologist has to face two main problems: monitoring the insertion length of the catheter and choosing the appropriate caliber of the device.

As regards the insertion length, the ideal position of the catheter tip is at the junction of the right atrium and inferior vena cava (IVC), in the subdiaphragmatic vestibulum outside the cardiac silhouette, and pointing to the right side of the base of the heart (1). A UVC follows the umbilical vein and traverses the central part of the left portal vein (Rex segment) into the ductus venosus (DV) to reach the IVC. It is fundamental to place the UVC correctly to avoid serious complications. If the catheter tip is too deep in the heart, there is a risk of cardiac arrhythmia, intracardiac thrombosis, myocardial perforation, pericardial effusion, or tamponade. Conversely, if the tip is low in the portal vein, it can lead to portal vein thrombosis (PVT), portal hypertension, and liver necrosis (2).

In recent years, there has been a constant improvement in the insertion and positioning technique of central vein occlusions (CVO), thanks to the increasingly widespread use of the tip navigation and location ultrasonographic (US) technique. Indeed, echocardiography allows direct visualization of the cavo-atrial junction through a parasagittal subcostal view, making this technique superior to chest radiography in determining the position of the tip of the UVC (3–6). In addition, real-time bedside use of US during umbilical vein catheterization is a promising technique for the rapid and accurate placement of the catheter (7–10).

Despite these technical improvements, to our knowledge, there is a knowledge gap in choosing the appropriate caliber of UVC. A normal practice is to use a 3.5-French catheter for babies weighing less than 1.5 kg and a 5-Fr catheter for bigger babies, as suggested by the main manuals used in neonatology (11–13). A recent review article suggests using 3.5-Fr catheters for infants weighing less than 3.5 kg and 5-Fr catheters for neonates weighing more than 3.5 kg, but no references are provided to support this recommendation (14). The choice of the right caliber of UVC is essential because it is well known that thrombosis risk significantly increases with increasing catheter size (15, 16). In addition, mathematical and experimental models demonstrate blood flow reduction because of the presence of a catheter in a vein. Consequently, it is suggested that the external diameter of the catheter should be equal to or smaller than one-third of the internal diameter of the vein (17, 18) or less than 45% (19).

Few data are available regarding the diameter of the veins of newborns (20). Even fewer data are available about the IVC diameter: current studies concern the ultrasonographic assessment of hydration status or the assessment of central venous pressure measurement in correlation with the IVC diameter (21–23). From what we know, there are no studies on the US identification of the UVC size according to the diameter of the vein.

In this prospective study, we aimed to evaluate the IVC diameters in a cohort of newborns weighing 2–4 kg and investigate the correlation between newborn weight and IVC diameter. The secondary outcome was to evaluate the concordance between the measures carried out by two investigators.

## Methods

In this prospective cross-sectional study, we performed the US evaluation of the IVC diameter in neonates weighing 2–4 kg.

The study was conducted between June 2019 and January 2021 in IRCCS Burlo Garofolo, Italy. The Bioethic Committee approved the study protocol.

Healthy newborns in the nursery ward and newborns admitted to our NICU with a weight of 2–4 kg were enrolled after obtaining informed written consent from parents. The exclusion criteria included hemodynamic instability, known vascular malformations, and major congenital malformations.

US scans were performed by a Logiq E9 Ultrasound Unit (GE Healthcare) using an S4-10 microconvex probe. Each neonate was placed in a supine position, and stress-reducing measures were used if needed. A transducer was placed over the subxiphoid region, avoiding abdominal compression, and a longitudinal image of the IVC was obtained. The maximal IVC anteroposterior diameter was measured in two dimensions (B-mode) from the inner wall to the inner wall (see Supplementary Figure S1). We followed the guidelines on echocardiographic chamber quantification published in 2015 by the American Society of Echocardiography, which recommends measuring the maximum IVC diameter from the subcostal view with the IVC displayed along its long axis. The diameter should be measured approximately 1–2 cm caudal to the junction of the IVC and the ostium of the right atrium (24).

Two adequately trained neonatologists (CB and FG) performed the scan consecutively, blinded to each other's results. Each examiner obtained multiple measurements of the vessel (at least two), and the highest value of the two was recorded.

*The primary outcomes* of this study were to evaluate the IVC diameter in a cohort of newborns and the correlation between newborn weight and IVC diameter.

To better evaluate a possible association between newborns’ weight and IVC size, neonates were divided into two groups based on their weight at the moment of the US examination: 2.0–2.99 and 3.0–4.0 kg. The categorizations of baby weight were established before starting the study based on our usual NICU population to obtain comparable categories.

*The secondary outcome* was to evaluate the concordance between the measures carried out by the two investigators.

Categorical data were presented as numbers and percentages, and continuous data were presented as medians, interquartile ranges (IQRs), and ranges. The Bland–Altman plot and the intraclass correlation coefficient (ICC) were used to evaluate the agreement between operators. The differences in the IVC diameter between weight groups, sex, and IV infusion were evaluated using the non-parametric Mann–Whitney test. The use of non-parametric tests is justified by the non-normal distribution of data, evaluated both visually and using the Kolmogorov–Smirnov test. The differences with a *p*-value of <0.05 were considered statistically significant. Analysis was performed using SPSS version 23 (IBM, New York, USA).

## Results

A total of 143 neonates were enrolled between June 2019 and January 2021, including 59 neonates (41.3%) in the 2.0–2.99 kg group and 84 (58.7%) in the 3.0–4.0 kg group. In total, 49.9% of the sample were female (*n* = 70). The median weight at the time of the US examination was 3.1 kg (IQR: 2.6–3.5), while the median birth weight was 3.2 kg (IQR: 2.7–3.5). All the US examinations were performed within the third day of life; specifically, 60 patients (42.0%) were examined within the first postnatal day, 43 (30.0%) within the second, and 40 (28.0%) within the third.

The median gestational age at birth was 39 weeks (IQR: 38–40). Patients were mainly healthy term infants admitted to the nursery ward, with 23 preterm babies. Of the 143 patients, 12 (8.4%) received IV infusion at the time of US examination.

**Figure 1:**
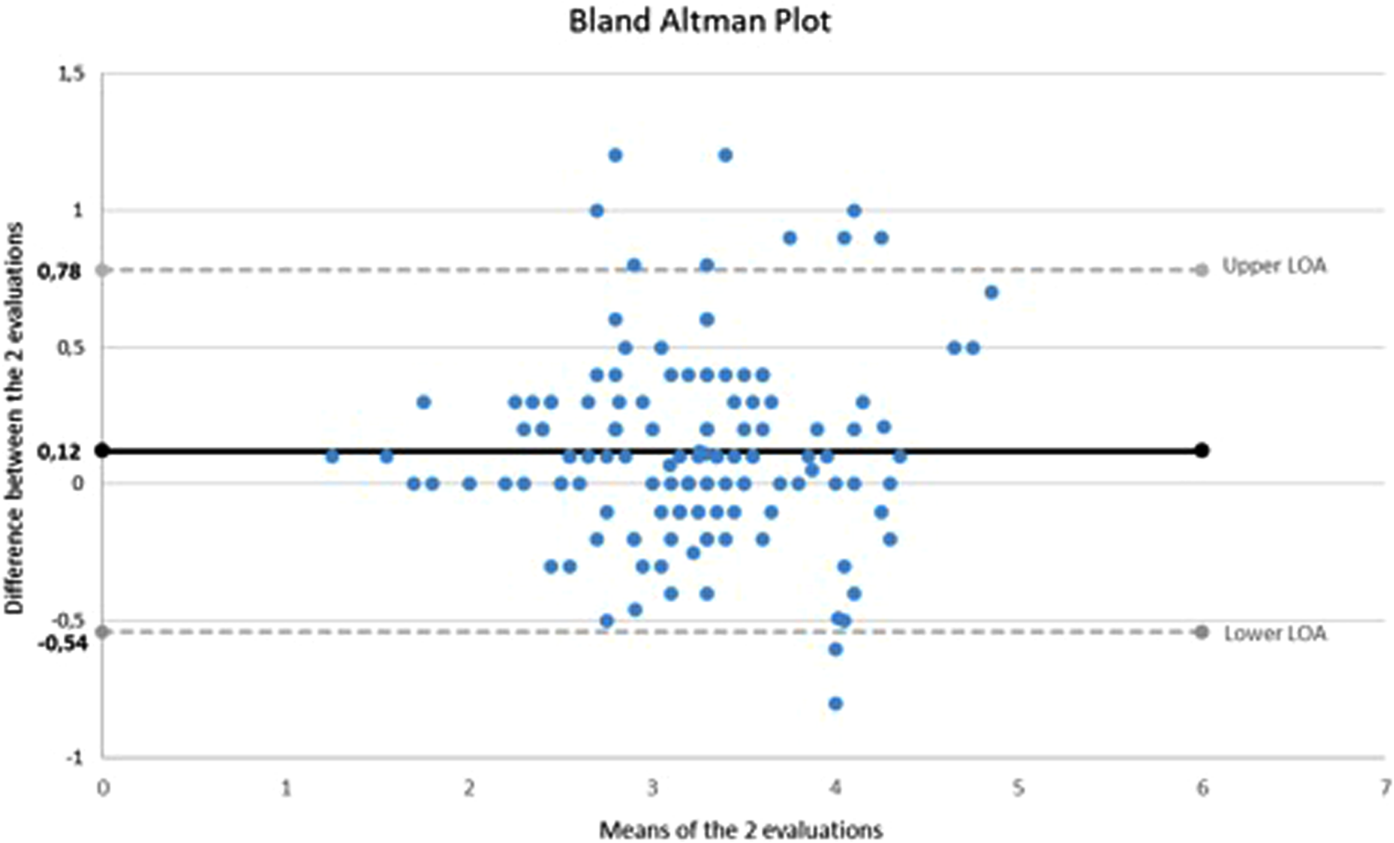
Bland–Altman plot. LOA, 95% limit of agreement.

The IVC median diameter measured by the first examiner was 3.3 mm (IQR: 2.9–3.7; range: 1.3–5.2) and by the second examiner was 3.2 mm (IQR: 2.7–3.5; range: 1.2–4.5). Figure 1 shows the Bland–Altman plot comparing the evaluations performed by the two examiners. The 95% limits of agreement (−0.54 to 0.78) include 93.3% (132/143) of the difference scores. The ICC was 0.93 (95% CI: 0.90–0.95).

There were no differences in IVC diameter by sex (*p* = 0.7 for both examiners) and IV infusion (*p* = 0.8 for both examiners).

**Figure 2:**
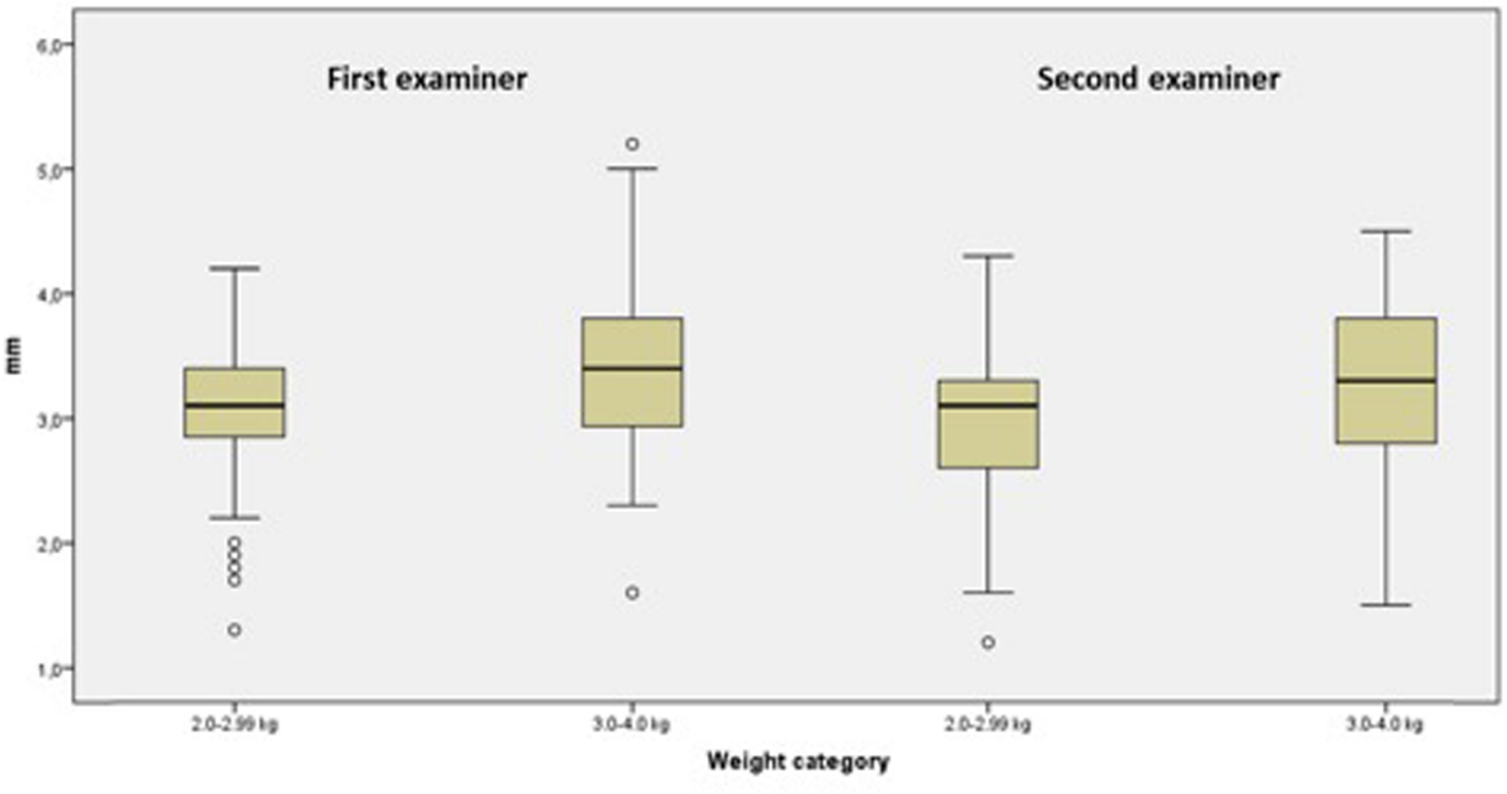
IVC median diameters measured by examiners 1 and 2 according to newborn weights.

After dividing the patients into two groups according to their weight at the time of examination (2.0–2.99 and 3.0–4.0 kg), the IVC median diameters measured by examiner 1 were 3.1 mm (IQR range: 2.8–3.4; range: 1.3–4.2) and 3.4 mm (IQR: 2.9–3.8; range: 1.6–5.2) (*p* = 0.003) and the IVC median diameters measured by examiner 2 were 3.1 mm (IQR: 2.6–3.3; range: 1.2–4.3 mm) and 3.3 mm (IQR: 2.8–3.8; range: 1.5–4.5 mm) (*p* = 0.004), respectively, for the two groups (Figure 2).

**Figure 3:**
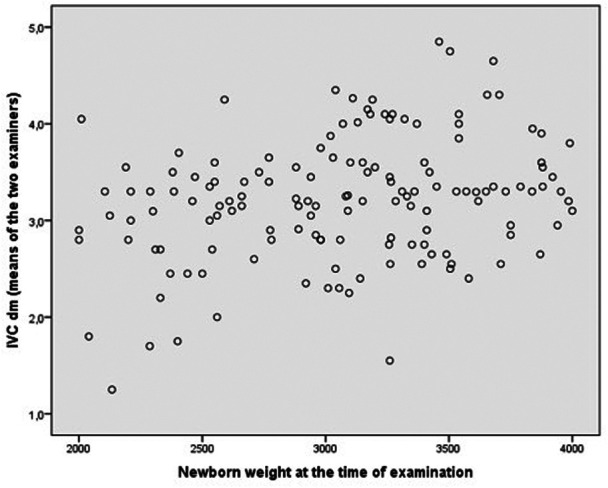
Relation between newborn weight at the time of examination and IVC diameter (mean value of the two examiners).

Figure 3 shows the relationship between newborn weight at the time of examination and IVC. Spearman's correlation coefficient was relatively low (rho = 0.25).

Considering the actual number of patients enrolled, means and standard deviations, and a precision of 5%, the study power was 0.70 for the 2–2.99-kg group and 0.76 for the 3–3.99-kg group.

## Discussion

Current guidelines suggest a catheter-to-vein diameter ratio equal to or smaller than one-third (17) or less than 45% (19) to reduce the risk of thrombosis.

Indeed, thrombosis is a well-known complication of central venous accesses, and the reported incidence of UVC-related thrombosis varies greatly due to the differences in study design and methodology: it is reported to occur in 3%–33% of infants after umbilical vein catheterization (13). Recently, although a much higher incidence was reported, the prospective study of Dubbink-Verheij et al. (25) found a 75% incidence rate of thrombosis, with 17% of clots involving the right atrium/IVC junction. By performing contrast venography, Roy et al. (26) similarly found 50% of the thrombi in the IVC, 29% in the right atrium, and 21% in the DV, with an overall incidence rate of UVC-related thrombosis of 30%. From these data, IVC seems to be the most involved structure in this complication.

Catheter-related PVT has been described as a rare event but is increasingly recognized, thanks to the increased use of US evaluations when the UVC is in place and after its removal. The reported incidence rate varies from 2.2% to 43% because of the differences in study design and methodology. UVC can cause thrombosis with different mechanisms: direct damage to vessel walls, disrupted blood flow, infusion of substances damaging endothelial cells, and introduction of a foreign thrombogenic surface (14).

The traditional risk factors of thrombosis are summarized by Virchow's triad, namely stasis, vascular injury, and hypercoagulability. Intravascular catheters can impact this triad by reducing the vein's blood flow (stasis), damaging the endothelium, and introducing a thrombogenic surface. Also, neonates are at a high risk of thrombosis because they have underdeveloped clotting mechanisms, small vessel diameter, and often underlying diseases such as perinatal asphyxia, hypovolemia, septicemia, polycythemia, or congenital cardiac disease (27). Studies described that thrombus formation in the UVC route could lead to thrombocytopenia, persistent sepsis, liver damage, portal hypertension, symptoms of right heart failure, and pulmonary embolism.

Although newborns have risk factors for thrombosis, especially those with UVC, no studies evaluate the size of the vessels crossed by UVC.

Ideally, a pre-procedural US scan of the veins has to be performed before any US-guided cannulation (18): we could identify the anatomic variants (such as azygos continuation or persistent right umbilical vein), evaluate the vascular flexion running from the portal sinus to the DV (useful aligning maneuver of the umbilical vein with the DV in a straight line under US guidance) (28), and measure the vein through which the catheter runs.

When a UVC is positioned, it is inserted into the unpaired vein of the umbilicus until it reaches the umbilical recess (UR) and should, if correctly positioned, reach the IVC through the DV. The catheter occurs for its greater length in the lumen of the DV, a vessel mostly virtual and, therefore, difficult to sample. With regard to the DV diameter, Zytoon et al. calculated the reference ranges for the DV diameter in the 690 fetuses, showing a parabolic curve with the first to ninety-ninth percentiles. It ranged from 0.06 to 0.16 cm with an average of 0.11 in the first trimester, from 0.08 to 0.18 cm with an average of 0.15 cm in the second trimester, and from 0.13 to 0.23 cm with an average of 0.16 cm in the third trimester (29). The decrease in diameter of the umbilical vein (UV) from the UR into the ductal vein is well known. This narrowness is caused by a connective tissue called the “border strip” and could lead to catheter malpositioning, depending on the size and flexibility of the catheter. Due to the high variability of measurement of the DV, we contemplated that the IVC diameter could be the most reliable and easily reproducible measurement.

Furthermore, a US-based study of the IVC is a feasible practice, and the bedside use of US for tip navigation and tip location is increasingly used in the NICU during the placement of central venous access devices. In detail, we chose the subcostal view (longitudinal view) since it allowed a rapid visualization of the vessel and was proved equally reliable to M-mode. During the examination in the longitudinal plane, the IVC anteroposterior diameter may be underestimated when the US beam goes through the vessel in an off-centered plane. Therefore, we considered the highest measure of the diameter (22).

Our study has several strengths. First, we examined a large neonatal population. In addition, we performed all the US examinations within 72 h of life because inserting an UVC after the third day of life is neither common nor feasible in clinical practice. Last but not least, two blinded examiners performed the ultrasonographic assessment, unlike the other available studies.

Concerning the concordance between the measures carried out by the two investigators, the interval in the vertical axis of the Bland–Altman graph (difference between the two evaluations) appears to be quite broad. However, the mean difference between evaluations is 0.12 mm, and 93% of the observations fall inside the 95% CI lines. The ICC suggests good agreement between evaluators.

There are several limitations to our study. First, we did not measure the DV and the portion of the IVC in which the catheter runs. Ideally, these measurements should have been performed. However, the natural involution of the DV, mostly a virtual vassel, is to close over time through thrombosis. Regarding the portion of the IVC measured, we chose the easiest and most reproducible US view, as close as possible to the point where the DV joins IVC. Indeed, an improvement point would be to measure the VCI closer to the subdiaphragmatic vestibulum using a different US view by trained operators (complementary windows such as the apical four-chamber view and the parasternal short-axis view).

Furthermore, in our study, as per protocol, we did not evaluate the IVC diameter in very low birth weight (VLBW) newborns, a category that requires UVC insertion more frequently. In this group of babies, UVC catheterization is often an emergency issue, and it would not be realistic to have the IVC diameter measured by both the examiners involved in the study before catheter insertion.

In conclusion, we believe that ultrasonography should be routinely performed to optimize UV catheter size choice, as it is already performed for the cannulation of the other central veins (27). Based on Spearman's rank correlation in evaluating the association between newborn weight and IVC, we found a low correlation. Therefore, in our study, it is impossible to identify a cutoff weight to choose a UV catheter. Therefore, measuring IVC may be a recommended step prior to inserting a UV catheter. Assuming that the catheter should be one-third of the vessel diameter (or catheter-to-vessel ratio <45%) to decrease the risk of thrombosis, we suggest that the IVC diameter of 3.5–5 mm requires a 3.5-Fr (1.15-mm) catheter and the IVC diameter of more than 5 mm requires a 5-Fr (1.6-mm) catheter.

In our study, although we did not find a correlation between the weight and size of the IVC, our result supported the recent indications in the literature regarding the choice of the 3.5-Fr catheter even in newborns under 3.5 kg (14).

Our assumptions on catheter size need to be verified. As far as we know, no studies focused on comparing the incidence of thrombosis using a 3.5-Fr vs. a 5-Fr UV catheter.

It will be interesting to generate data about this common procedure. These data may provide the desired evidence to guide clinicians on the best UV catheter size choice, which in turn could reduce the incidence of thrombosis.

## Data Availability

The raw data supporting the conclusions of this article will be made available by the authors, without undue reservation.
