# Supplementary material for: Ultrasonographic measurements of the inferior vena cava diameter in newborns: is it a useful tool for choosing an umbilical venous catheter?
Source: Front Pediatr. 2023 Nov 17;11:1268622. doi: 10.3389/fped.2023.1268622 (PMC10690934; doi:10.3389/fped.2023.1268622)
Supplement: Supplementary file 1 [file Table1.docx]

Supplementary Material

Inferior vena cava diameter ultrasound measurements in newborns: is it a useful tool for choosing the umbilical venous catheter?

**Galdo F^1^, Trappan A^1^, Cossovel F ^2^, Rodriguez-Perez C ,^4^ Ronfani L ^5^, Montaldo P^6,7^, Bibalo C^8,^ Travan L ^1^, Risso FM ^4^**

^1^ Neonatal Intensive Care Unit, Institute of Child and Maternal Health, IRCSS Burlo Garofolo, Trieste, Italy.

^2^ University of Trieste, Trieste, Italy

^4^ Neonatology and Neonatal Intensive Care Unit, ASST Spedali Civili, Ospedale Dei Bambini, Brescia, Italy

^5^ Clinical Epidemiology and Public Health Research Unit, Institute for Maternal and Child Health - IRCCS "Burlo Garofolo", Trieste, Italy

^6^ Department of Neonatal Intensive Care, Università degli Studi della Campania Luigi Vanvitelli, Naples, Italy

^7^ Department of Brain Sciences, Centre for Perinatal Neuroscience, Imperial College, London, UK

^8^ Azienda Sanitaria Universitaria Giuliano Isontino, Triese, Italy

*** Correspondence:**Francesca Cossovel
[francesca.cossovel@gmail.com](mailto:francesca.cossovel@gmail.com)

**Supplementary figures**


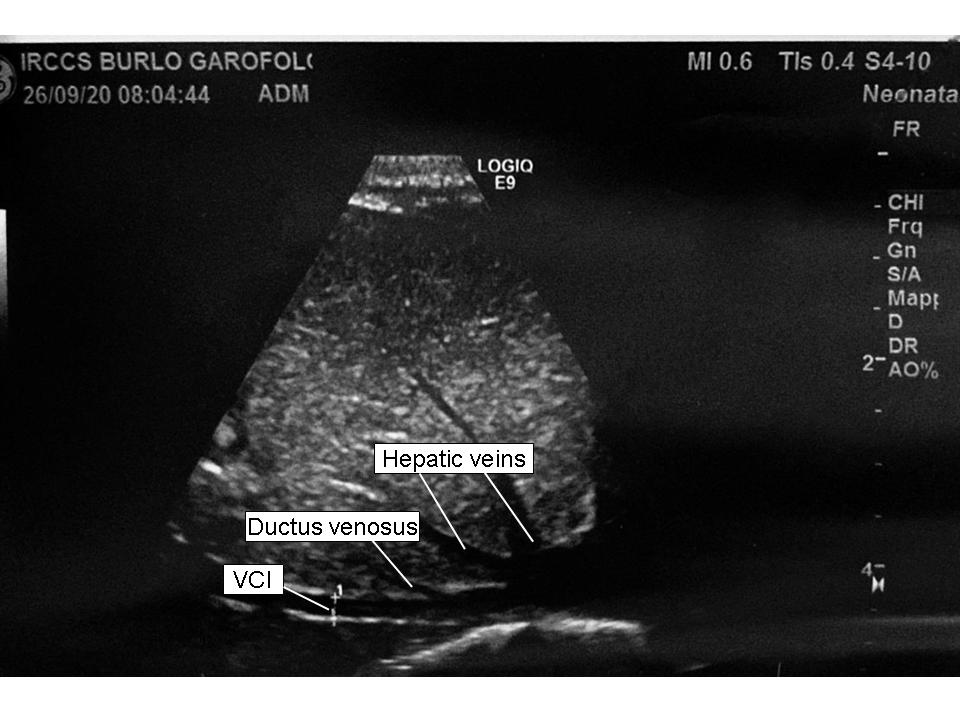


## Supplementary Figure 1: The transducer was placed over the subxiphoid region, avoiding abdominal compression, and a longitudinal image of the IVC was obtained. The maximal IVC anteroposterior diameter was measured in two-dimensional (B-mode), from inner wall to inner wall (see supplementary figure 1). We followed the Guidelines on echocardiographic chamber quantification published in 2015 from the American Society of Echocardiography which recommend that the maximum IVC diameter was measured approximately 1–2 cm caudal to the junction of the IVC and the ostium of the right atrium.
